# Association between dental agenesis and facial morphology. A cross-sectional study in France

**DOI:** 10.1371/journal.pone.0314404

**Published:** 2024-12-06

**Authors:** Damien Brézulier, Pierre Raimbault, Sylvie Jeanne, Tiphaine Davit-Béal, Guy Cathelineau

**Affiliations:** 1 CHU Rennes, Pôle Odontologie, Univ Rennes, Rennes, France; 2 CHU Rennes, Inserm, Centre d’Investigation Clinique de Rennes (CIC 1414), Rennes, France; 3 ISCR UMR 6226, Univ Rennes, Rennes, France; Universidade Federal Fluminense, BRAZIL

## Abstract

**Purpose:**

Knowing the features of dental evolution and facial morphology, marked by an increase in the prevalence of agenesis and a tendency towards verticalization of the face, the main objective of this cross-sectional observational study was to assess the correlation between the occurrence of agenesis (of 3^rd^ molars or other teeth) and facial morphology in the French population.

**Methods:**

The study was conducted at the University Hospital of Rennes, France, from June 2022 to October 2022. Patients aged 12–18 years who underwent a global orthodontic assessment were included. Data collected from medical examinations, panoramic, anteroposterior, and lateral x-rays were analyzed for cephalometric and dental features. The association between morphological parameters and agenesis of wisdom teeth or other teeth was assessed by univariate and multivariate analysis.

**Results:**

The study included 259 patients, of whom 89 presented agenesis. Logistic regression analyses identified several morphological parameters associated with agenesis. In the multivariate model, a negative correlation was found between tooth agenesis and FMA (OR = 0.85; p < 0. 001), facial axis (OR = 0.92; p = 0.040), and a positive one with SNB (OR = 1.17; p = 0.023), ANS-Xi-Pm (OR = 1.12; p = 0.013).

**Conclusion:**

This study highlights the correlation between agenesis occurrence and specific cephalometric parameters in the French population. The findings suggest that transverse constriction of the maxilla, facial divergence, and anterior projection of the chin symphysis are associated with agenesis.

**Trial registration number:** Opinion n°22.103, dated 06.04.2022.

## Introduction

The oldest fossil attributed to the Homo genus is a fragmentary mandible dated to around 2.8 million years ago. Today, the beginning of the Homo lineage is established with certainty at 2.5 million years B.C. [1]. However, the sequence of different human species over the last 2.5 million years remains a subject of debate among specialists. There is no consensus on phylogeny. The expansion of the cranial cavity, the reduction of the bimaxillary prognathism, the reduction of the dentition, the descent of the larynx and the reduction of the digestive tract are evolutionary features that stand out. They are in line with the evolution of the lineage, marked by a straightening of the posture, verticalization of the face and limited growth of the facial mass, particularly in the middle section. The 32-tooth permanent dentition was already present in our close ancestor Homo Habilis [2]. Agenesia, the reduction of the dentition due to failure of development, is one of the first eruption anomalies of the adult dentition. Its etiology remains unclear, although genetic responsibility has been confirmed [3].

In this ever-changing context, different types of agenesis have been identified. Although they mainly affect the so-called "end-of-series" teeth, i.e. lateral incisors, second premolars and third molars, their prevalence is constantly increasing [4]. Teeth act as functional units that stimulate local bone growth. In this sense, their presence or absence seems to condition the future of the skeleton. This occurs both directly, as the alveolar process is forced to grow, and indirectly, as occlusion forces the facial structure to grow along the lines of masticatory stress [5]. In fact, the literature is full of examples of bi-maxillary retrognathism, reduced maxillary and mandibular length, and receding chins [6]. However, some cases of prognathism have been described [7]. Since the evolution of the species involves retrognatism or a reduction in the dental formula, we can ask to what extent these parameters are associated. These dental and skull changes have direct implications for orthodontics, as it has already been demonstrated that understanding the evolution of human teeth is essential for developing effective strategies for preventing and treating malocclusions [8, 9].

Previous work has shown that third molar agenesis is associated with a reduction in Jarabak’s gonial angle and upper gonial angle, characteristic of patients with a more horizontal or brachyfacial skeletal pattern. However, they were limited to the impact of wisdom teeth [10, 11]. The hypothesis tested here is that skeletal and alveolar cephalometric factors would correlate with dental factors, beyond the third molars.

The aim of this retrospective study was to evaluate the association between the occurrence of dental agenesis and variations in facial skeletal growth in sagittal, vertical and transverse dimensions in young subjects.

## Materials and methods

### Study design and participants

A cross-sectional observational study was conducted according to the STROBE (Strengthening the Reporting of Observational Studies in Epidemiology) recommendations. The study was approved by the Ethics Committee of the University Hospital of Rennes on April 6th, 2022 (opinion n°22.103). In accordance with French regulations, the parents or legal representatives of the minors were informed orally and in writing that their healthcare data could be re-used for retrospective studies. They signed this information at the initial consultation. When the data were reused for the study, the parents of the minors were informed of the process by letter. They were sent an information letter including data management and anonymization. At the end of this campaign, the files of children whose parents had received detailed information by information letter and had responded unfavorably to inclusion were not retained. This information was added to the medical file. Data was collected between June 2, 2022 and October 31, 2022. To ensure anonymity, a first file listed the patients included. It indicated the correspondence between patient identity and file number. It should be noted that only one of the authors (PR) had access to this file. This file was entrusted to the Research and Innovation Department. A second file was used to collect data for each anonymous file number. All relevant data are within the manuscript and its Supporting Information files (S1 Appendix).

Patients who consecutively consulted for a global orthodontic assessment at University Hospital of Rennes between May 2018 and September 2022 were included in the study. The data collected came from the medical examination, panoramic, anteroposterior (AP) and lateral x-rays. Inclusion criteria were: (1) patients aged 12 to 18 years old, (2) with a complete medical record (3) adequate quality lateral and frontal cephalometric radiograph in maximal intercuspation, depicting a reference ruler for magnification measurement, (4) adequate quality panoramic radiographs for identification of missing teeth. Exclusion criteria were (1) incomplete medical record including lack of x-ray imaging, (2) unreadability of imaging, (3) intervention known to influence craniofacial morphology, such as orthodontic treatment (4) history of tooth extraction, (5) pathology or syndrome influencing facial growth, (6) more than 6 permanent teeth missing, (7) agenesis involving both wisdom teeth and other teeth.

### Studied variables

The data collected from the panoramic radiographs described the number of agenesis and the teeth involved.

**Fig 1:**
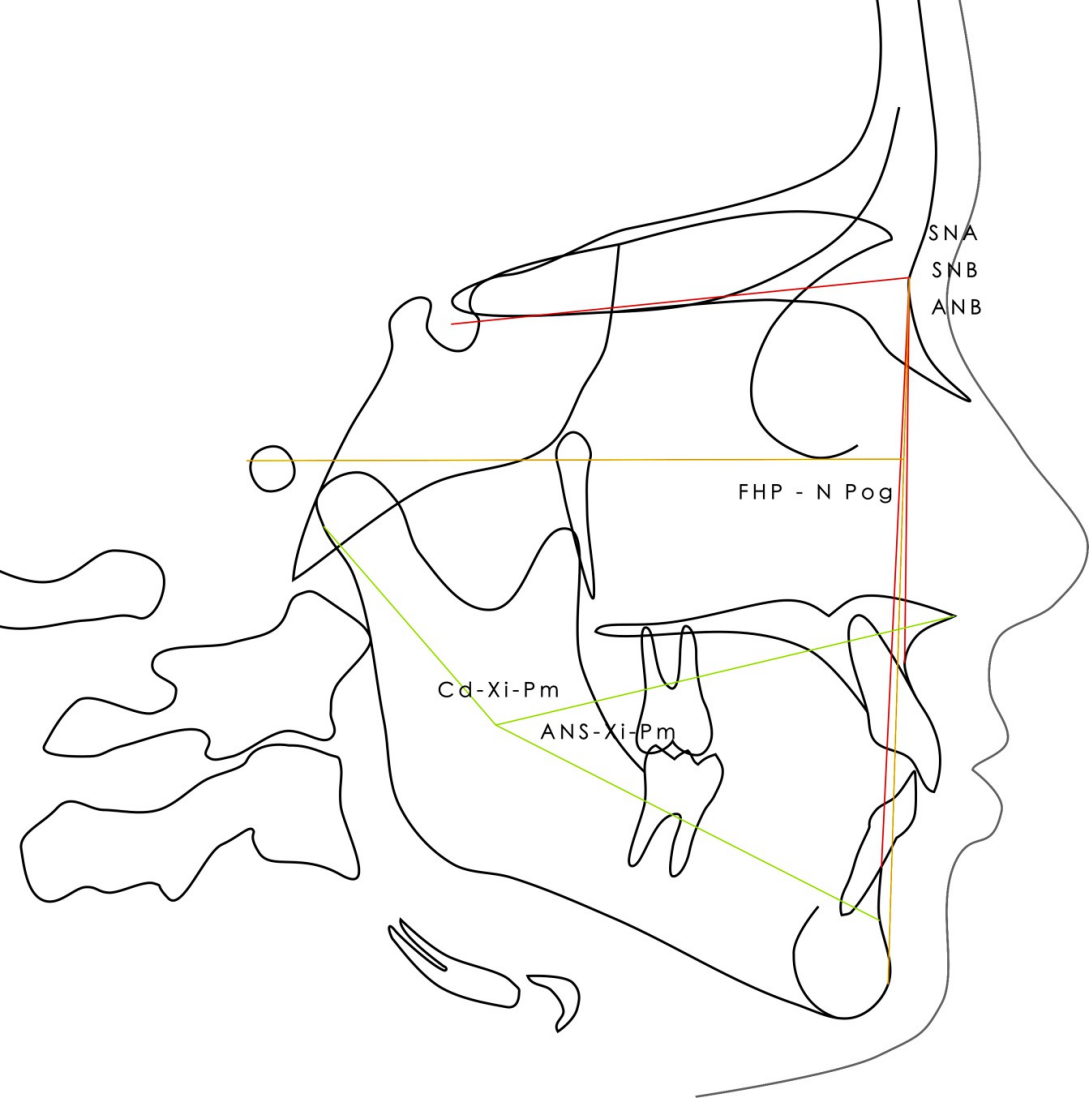
Cephalometric profile. Illustration of the planes and angles used in profile analysis.

Lateral x-rays were used for sagittal and vertical description. SNA, SNB, ANB and FMA angles were taken from Tweed analysis. The position of the chin (facial depth angle: Frankfort Horizontal Plane to Nasion-Pogonion, FHP-NPog), the morphology of the mandible angle (Cd-Xi-Pm), the divergence of the maxillae (Anterior Nasal Spine-Xi-Pm, ANS-Xi-PM) and finally the facial axis were taken from the bio-progressive analysis of Ricketts (Fig 1). Each lateral x-ray was manually analyzed using the cephalometric analysis module of Logos_W software (Liffré, France).

**Fig 2:**
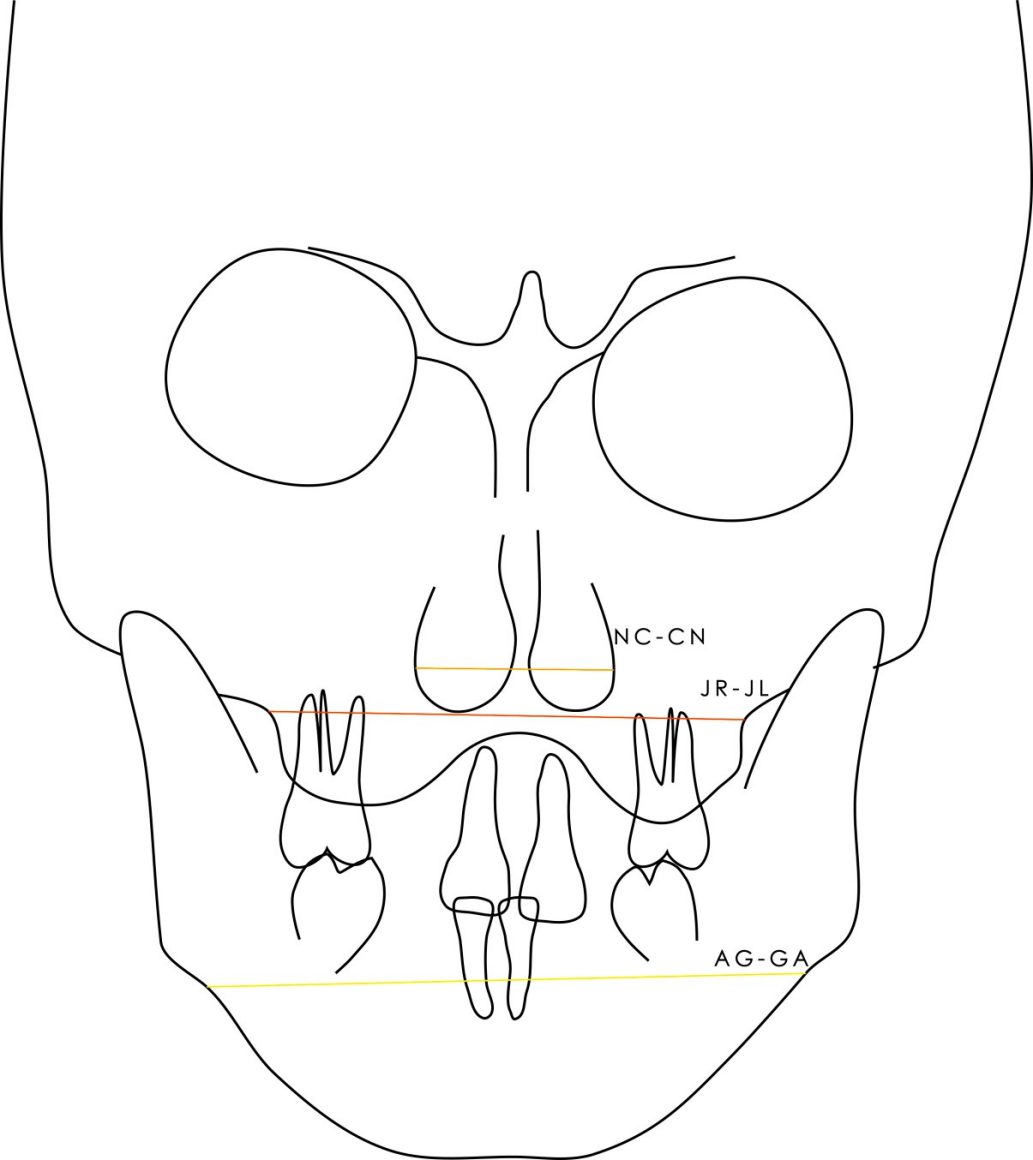
Frontal cephalometry. Illustration of the frontal measurements.

On frontal x-rays, width of piriform aperture (Ricketts’ NC-CN), distance between the intersection of the lateral surface of the maxillary tuberosity and the zygomatic process, left and right (JR-JL), and distance between the highest points of the left and right ante-gonia notches (AG-GA) measurements described the transverse dimensions of the piriform aperture, maxilla, and mandible (Fig 2).

### Statistical analysis

Data were compiled in a Microsoft Excel® spreadsheet. Statistical analysis was performed using RStudio® software version RStudio 2023.06.1+524 (RStudioTeam) in R language version R 4.3.1 (RCore Team). Descriptive statistics were calculated for the variables of interest. Descriptive results are reported as mean and standard deviation. Student’s t-test or ANOVA were performed to compare quantitative values. Univariate and multivariate logistic regression analyses were performed to identify morphological parameters associated with agenesis. The co-variates from the univariate analyses were fed into top-down multivariate stepwise regression analyses based on Akaike Information Criterion (AIC). The complete and reduced models were compared by chi-squared test. The Hosmer-Lemeshow goodness of fit test assessed the predictive value of binary logistic regressions. A p-value < 0.05 was considered significant. Intraclass correlation coefficient (ICC) determined intra-rater reproducibility for quantitative values. It was calculated on 10 cases, with readings taken at 1-month intervals.

## Results

### Intra-rater reliability

The ICC measurement yielded an 0.827 for quantitative measures, i.e. a good reproducibility.

### Descriptive analysis of the sample and frequency of agenesis

**Fig 3:**
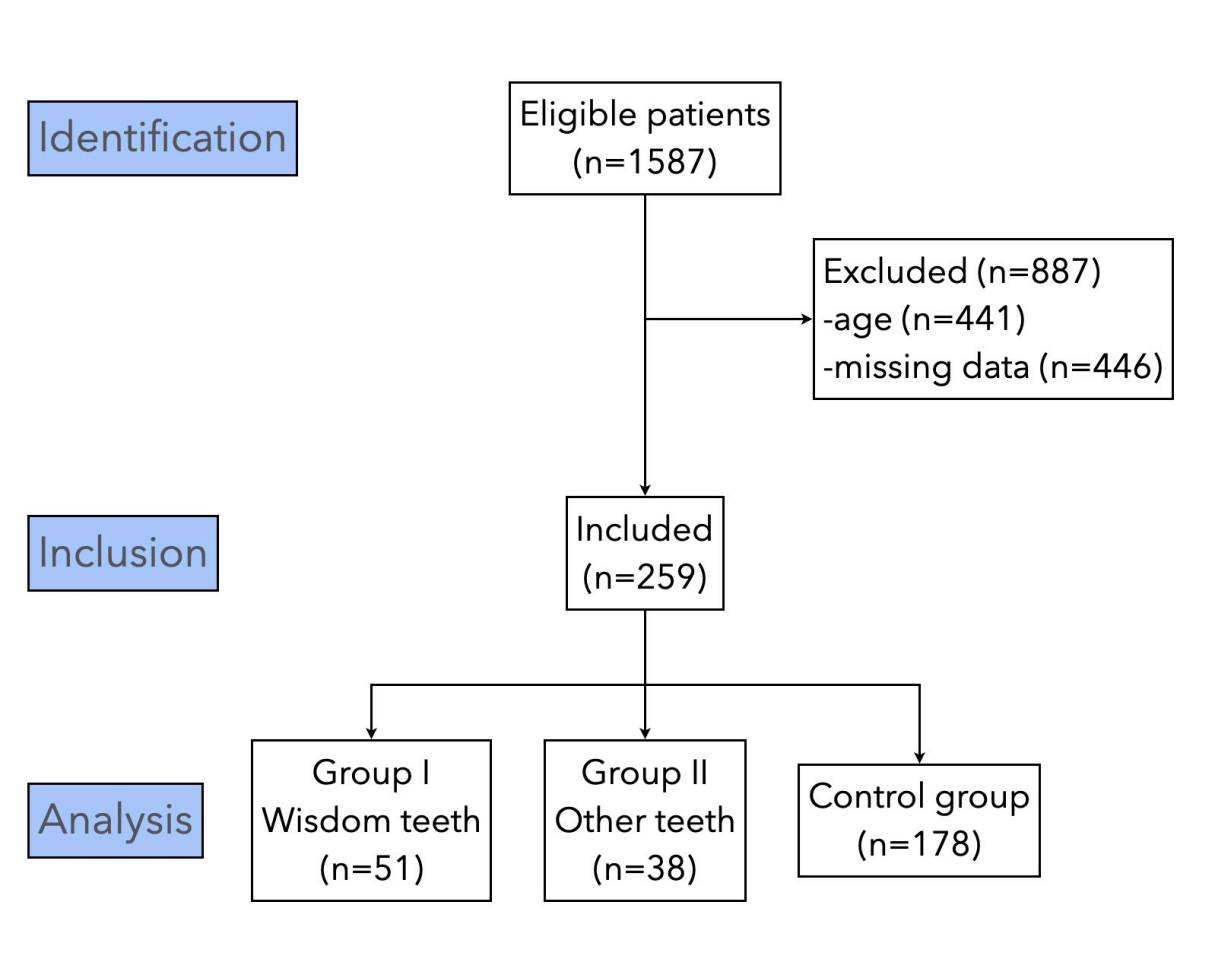
Flow chart. Study flow chart according to STROBE recommendations.

A total of 1587 records were reviewed, of which 1146 matched the target ages. 887 were excluded leaving 259 patients included (Fig 3). The final sex ratio was 1:1 and the mean age was 13.7 ± 1.6 years, with no difference between boys and girls (p = 0.54). Agenesia was detected in 89 patients (34.4%). Specifically, 51 (19.7%) for wisdom teeth only (group I) and 38 (14.7%) if other teeth were considered (group II). For group I, 1 or 2 teeth were most often missing (n = 38; 74.5%). For group II, 1 or 2 teeth were missing for 22 patients (57.9%), 3 or 4 teeth for 7 patients (18.4%) and more than 4 teeth for 9 patients (23.7%).

### Transversal dimension

Piriform aperture widths were comparable, 27.9 ± 2.8 mm, 28.6 ± 3.2 mm and 29.0 ± 3.2 mm respectively for groups I, II and control (without agenesis), with no significant difference (p = 0.10). Mandibular dimensions were 80.1 ± 4.8 mm, 79.5 ± 5.3 mm and 80.0 ± 4.9 mm respectively for groups I, II and control (p = 0.72). Maxillary width was greatest in the control group, 62.2 ± 4.9 mm versus 60.8 ± 4.7 mm or 60.8 ± 4.7 mm for groups I and II, but without been significant (p = 0.090).

Univariate regression was then used to characterize the association between the occurrence of at least one agenesis and transverse width measurements (Table 1). In group I, the frequency of wisdom tooth agenesis decreased significantly with increasing width of the piriform aperture (OR = 0.9; CI = 0.81, 0.99; p = 0.04). No association was found for group II. Dental agenesis, whether of wisdom teeth or another tooth, was significantly negatively correlated with maxillary width compared with the control group (OR = 0.94; CI = 0.89, 0.99; p = 0.03).

**Table 1:** Analysis of transverse dimension parameters.

Ricketts’ measurements	Group I			Group II			All agenesis		
	OR^*1*^	95% CI^*1*^	p-value	OR^*1*^	95% CI^*1*^	p-value	OR^*1*^	95% CI^*1*^	p-value
NC-CN	0.90	0.81, 0.99	**0.040**	0.99	0.89, 1.11	0.87	0.92	0.85, 1.00	0.065
JR-JL	0.95	0.89, 1.01	0.13	0.95	0.89, 1.03	0.21	0.94	0.89, 0.99	**0.030**
AG-GA	1.00	0.94, 1.07	0.88	0.97	0.90, 1.04	0.42	0.99	0.94, 1.04	0.63

Univariate regression analysis of agenesis occurrence in relation to transverse dimension parameters in groups I, II and for all agenesis.

^*1*^ OR = Odds Ratio, CI = Confidence Interval.

### Sagittal and vertical dimensions of the facial structures

Considering the sagittal dimension, there was no difference between group I, II and control (without agenesis) for SNA (p = 0.087), SNB (p = 0.31) and ANB (p = 0.14). Mean values were 83.7 ± 4.3°, 79.8 ± 4.3° and 4.0 ± 2.8° respectively. Instead, the Pog point projected further forwards in group II than in group I or control, with facial depth angle reaching 90.9° ± 4.3, 88.0° ± 3.9 and 88.4° ± 3.5 respectively (p < 0.001).

Concerning vertical dimension, facial divergence using FMA in group II was lower than in group I and control (p = 0.017), respectively 18.7° ± 5.6, 21.3° ± 6.3 or 21.7° ± 5.5. However, mandibular morphology was unrelated to agenesis, since Cd-Xi-Pm remained stable at 28.5° ± 5.9 and close to the norm of 26° (p = 0.7). The dental component of the lower facial height, angle ANS-Xi-Pm, was roughly equivalent in all groups, at 43.2° ± 4.8 (p = 0.26). Finally, the Ricketts facial axis followed the same trend, averaging 93.3° ± 5.3, with no difference between groups (p = 0.14).

In a subsequent step, univariate analysis looked for associations between the occurrence of agenesis and anteroposterior and vertical parameters (Table 2). In group I, the occurrence of missing wisdom teeth increased with maxillary retrusion seeing SNA (OR = 0.92; CI = 0.86, 0.99; p = 0.037). In group II, the occurrence of agenesis increased with decreasing facial divergence using FMA (OR = 0.91; CI = 0.86, 0.97; p = 0.006) and increasing projection of the pogonion point (OR = 1.20; CI = 1.09, 1.32; p < 0.001). No association was found for agenesis in general.

**Table 2:** Analysis of vertical and sagittal dimensions parameters.

Measurements	Group I			Group II			All agenesis		
	OR^*1*^	95% CI^*1*^	p-value	OR^*1*^	95% CI^*1*^	p-value	OR^*1*^	95% CI^*1*^	p-value
SNA	0.92	0.86, 0.99	**0.037**	1.05	0.97, 1.13	0.27	0.97	0.91, 1.03	0.35
SNB	0.97	0.90, 1.04	0.35	1.06	0.98, 1.14	0.17	1.01	0.95, 1.07	0.80
ANB	0.91	0.82, 1.02	0.10	0.95	0.84, 1.08	0.44	0.91	0.83, 1.00	0.053
FMA	1.00	0.95, 1.06	0.88	0.91	0.86, 0.97	**0.006**	0.96	0.91, 1.00	0.050
FHP N-Pog (facial depth angle)	0.94	0.87, 1.02	0.15	1.20	1.09, 1.32	**<0.001**	1.06	0.99, 1.13	0.11
Cd-Xi-Pm (angle morphology)	1.00	0.95, 1.05	0.93	0.98	0.92, 1.03	0.42	0.99	0.94, 1.03	0.50
ANS-Xi-Pm (oral cavity divergence)	1.05	0.99, 1.12	0.12	1.01	0.94, 1.08	0.83	1.04	0.99, 1.10	0.14
Ricketts’ facial axis	0.95	0.89, 1.00	0.073	1.04	0.98, 1.11	0.23	0.99	0.94, 1.03	0.54

Univariate regression analysis of agenesis occurrence in relation to vertical and sagittal parameters in groups I, II and for all agenesis.

^*1*^ OR = Odds Ratio, CI = Confidence Interval.

### Proposal of an association model between cephalometric factors and agenesis

**Fig 4:**
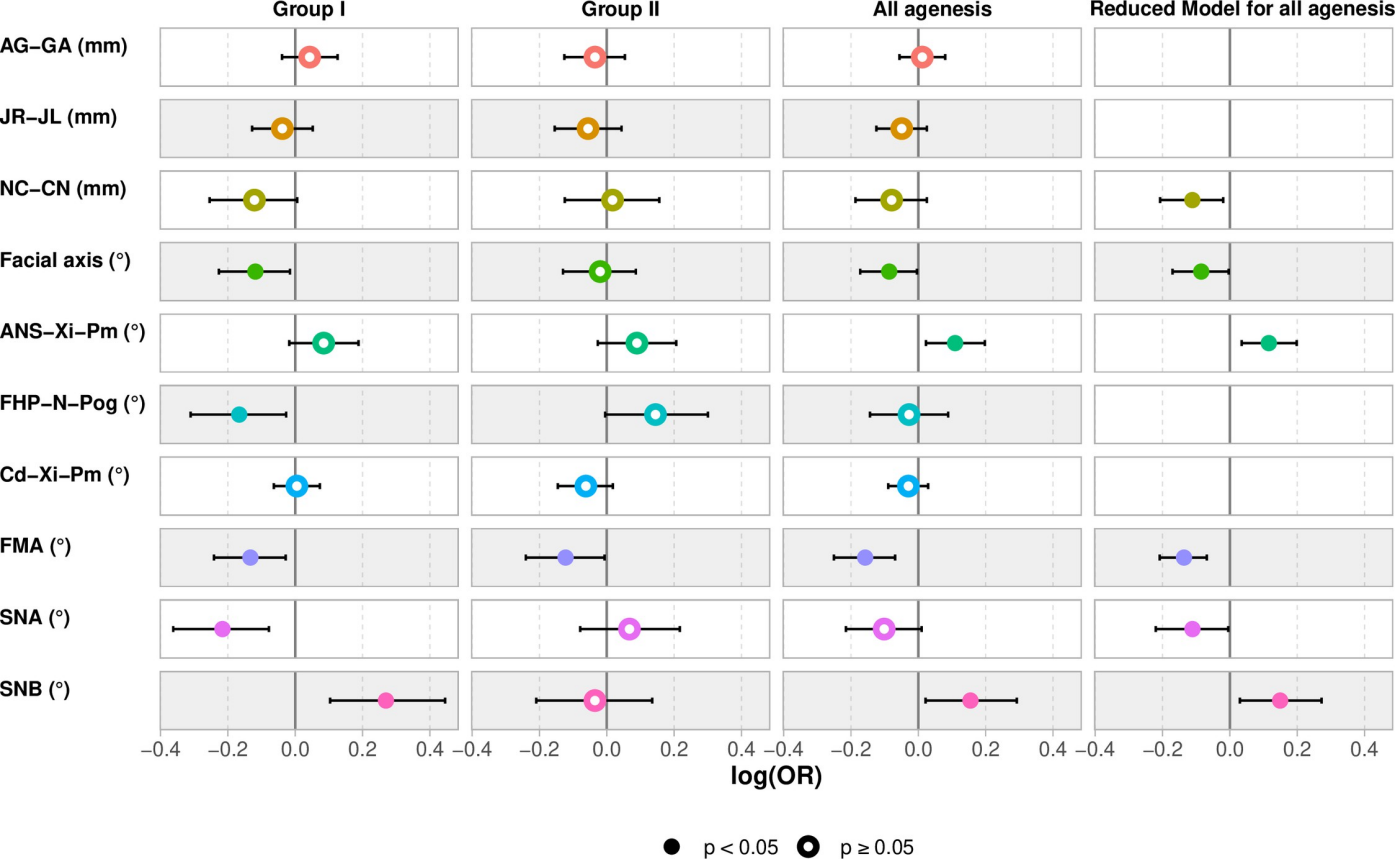
Multivariate regression model. Presentation of four models for the occurrence of agenesis according to various cephalometric parameters for groups I, II and all agenesis according to complete models, then proposal of a reduced model.

The above cephalometric factors were included in the multivariate analysis of agenesis occurrence (Fig 4). Given the risk of collinearity between SNA, SNB and ANB, ANB has not been included in the model.

For group I, the generalized regression model showed a negative association between wisdom tooth agenesis, increased facial axis (OR = 0.89; CI = 0.80, 0.98; p = 0. 024), facial depth angle (OR = 0.85; CI = 0.73, 0.97; p = 0.019), increased facial divergence (OR = 0.88; CI = 0.79, 0.97; p = 0.013) and SNA angle (OR = 0.81; CI = 0.70, 0.92; p = 0.002). It also highlighted a positive association with increased SNB (OR = 1.31; CI = 1.11, 1.56; p = 0.001). In group II, the occurrence of agenesis was linked only to the value of the FMA, facial divergence (OR = 0.88; CI = 0.79, 0.99; p = 0.038).

Considering the occurrence of agenesis without prediction of tooth type, the regression model negatively associated FMA (OR = 0.85; CI = 0.78, 0.93; p < 0. 001), facial axis (OR = 0.92; CI = 0.84, 0.99; p = 0.040), and positively SNB (OR = 1.17; CI = 1.02, 1.34; p = 0.023), ANS-Xi-Pm (OR = 1.12; CI = 1.02, 1.22; p = 0.013). The Hosmer-Lemeshow test concluded that the model had good predictive value (c^2^ = 12.19; p = 0.14).

Last, the stepwise top-down selection technique was used to simplify the model, generating a reduced model (p = 0.58). The reduced model demonstrated a negative association between agenesis, increased facial axis (OR = 0.92; CI = 0.84, 0.99; p = 0. 041), FMA (OR = 0.87; CI = 0.81, 0.93; p < 0.001), SNA angle (OR = 0.89; CI = 0.80, 1.00; p = 0.040) and NC-CN (OR = 0.89; CI = 0.81, 0.98; p = 0.017). It also revealed a positive association with increased SNB (OR = 1.16; CI = 1.03, 1.31; p = 0.014) and ANS-Xi-Pm (OR = 1.12; CI = 1.04, 1.22; p = 0.005). It had an AIC of 319.25 versus 324.37 for the full model. The Hosmer-Lemeshow test concluded that the reduced model had good predictive value (c^2^ = 7.11; p = 0.52).

## Discussion

Dental anthropology is an active field of research that studies the evolutionary aspects of tooth development: variations in number, size and morphology within populations. Tooth agenesis limited to a few specific teeth is common and often considered a normal variant. In one study on the issue, excluding 3^rd^ molars, there were no patients with more than two missing teeth [12]. This led us to limit cases to the lack of 6 permanent teeth. Cases of oligodontia were therefore not studied, as most of them are genetic diseases [13].

The permanent dentition is more frequently affected than the primary dentition. Excluding 3^rd^ molars, the prevalence of agenesis of permanent teeth varies from 1.6% to 9.6% in the general population. The prevalence found here was higher, at 14.7%. If wisdom teeth are included, the prevalence rises to 22.6% [14]. The higher prevalences we found are attributable to selection bias: population consulting an orthodontist. Even if wisdom tooth agenesis falls outside the definition of oligodontia, it is not trivial. They were already found in Homo sapiens 300,000 years ago [15]. The prevalence of missing third molars is thought to have risen to 20.8% today [16]. It could be due to genetic drift of the genus, with variations between different ethnic groups [17]. This is why we chose to study the two groups independently before mixing them.

The hypothesis tested here is that skeletal and alveolar cephalometric factors would correlate with dental factors and the main objective of this study was to assess the correlation between the occurrence of agenesis (of 3^rd^ molars, other teeth or overall) and facial morphology in the French population. An initial univariate analysis was supplemented by a multivariate model reduced by a top-down stepwise approach to identify correlations between the occurrence of agenesis and several explanatory and independent variables, namely cephalometric features. These correlations express the notion of a linear link between skeletal changes and the occurrence of missing teeth. The dichotomy into two groups enabled the identification of parameters specific to wisdom teeth. The number of parameters in the multivariate model is greater when only wisdom teeth are involved, with five parameters retained. In contrast, only one parameter was retained for the other group, including the other teeth, and this was the negative correlation with facial divergence.

Our results tended to indicate a negative correlation in favor of transverse constriction of the maxilla, both in terms of piriform aperture and zygomatic width in cases of agenesis. In the vertical dimension, they revealed a reduction in facial divergence, an anterior projection of the chin symphysis and a receding maxilla. However, no association was found between mandibular morphology and dental context. For instance, neither the mandibular angle, the Ricketts facial axis, nor the mandibular inter-angular width exhibited any correlation with the context of agenesis.

The observed increase in alveolar levels may be interpreted as an alveolar compensation mechanism attempting to limit the loss of lower facial height induced by the decrease in facial divergence. In this case, the lower height would be reduced by atrophy of the maxillary portion between the palatal plane and the base of the skull.

Taken together, these agenesia were rooted in a context of sagittal, vertical and transverse maxillary brachygnathia, associated with a more pronounced chin projection that could be explained by closure of the mandibular compass. This finding corroborated the tendency towards skeletal Class III found in the literature [18]. This is due to the posterior position of the maxilla. It may be suggested that hypodontia causes a lack of occlusal support, resulting in underdevelopment of the maxilla [7].

These results must be put into perspective in order to improve orthodontic treatment. For example, the risk of maxillary deficiency in cases of agenesis should be taken into account, and orthodontic mechanics should be implemented to avoid aggravating this tendency [19]. The use of temporary anchorage devices is therefore recommended to optimize outcomes of treatment.

These results and analyses should also be viewed in the light of potential biases in this study. For example, the type of missing teeth was not recorded, apart from the wisdom tooth/other tooth dichotomy. The group of agenesis other than wisdom teeth was therefore heterogeneous. This makes it difficult to determine how overall tooth agenesis is associated with reduced maxillary dimensions. These results could simply be due to the fact that most of the missing teeth in this cohort were maxillary teeth. Also, in this group, it is not possible to study an effect by tooth type, since some types may be too poorly represented. Finally, in view of the number of cases included, it did not seem relevant to us to stratify the study according to patient age, at the risk of losing power. Further studies on a larger cohort should be proposed.

Several hypotheses have been put forward to explain the link between agenesis and maxillary atrophy. The most studied in the literature is based on genetics. Several genes linked to dental agenesis in humans have been shown to regulate craniofacial bone morphogenesis. The Msx1 homeobox gene codes for a transcription factor that is highly expressed in bone during embryogenesis and postnatal development [20]. In humans, Msx1 mutations are associated with cleft palate and dental agenesis [21]. Experimentally, the effects of Msx1 on craniofacial bone morphogenesis have been shown to be significant [20]. Three genes are also associated with the non-syndromic form of human dental agenesis: Axin2, Msx1 and Pax9 [22]. Pax9 plays an essential role in craniofacial development [23]. Mice homozygous for a Pax9 deletion die soon after birth due to respiratory problems and show a wide range of developmental defects: secondary cleft palate, facial anomalies and complete anodontia with arrested tooth development at the bud stage [24].

Phylogenetic changes in dentition are correlated with functional adaptation. Although the genetic context is undeniable, it is becoming increasingly clear that epigenetic pressure is also responsible for agenesic phenotypes [25, 26]. This combination means that teeth and tooth-bearing bones evolve together. The reduction in the number of teeth is concomitant with the reduction in jaw size in human evolution, and is thought to be an ongoing evolutionary trend [27, 28]. Studies of primates, great primates and Homo sapiens have shown that Homo sapiens has a tendency to reduce the projection of the facial mass compared to its ancestors [29, 30]. The number of teeth decreases in parallel with these changes in the jaw skeleton [31]. Two theories have been advanced to explain the verticalization of the facial mass. The first is that verticalization is at the origin of bipedalism, an adaptation acquired by our ancestors [32]. For the second, the retreat of the maxillae is the consequence of the acquisition of bipedalism and nutritional changes leading to the Homo lineage descended from Australopithecus [33].

The genus Homo, which includes present-day humans, is distinguished by a reduced face, low skeletal sexual dimorphism, exclusive bipedalism for running, an advanced occipital foramen, an aptitude for running, and tool production. As with all other species, Homo sapiens is part of a constantly evolving lineage. This evolution is of particular interest as it depends on the interplay of genetic, epigenetic and environmental factors. This is evidenced by the verticalization of the face and the reduction of the dental formula.

## Conclusion

It is known that genus evolution is marked by a straightening of the posture, verticalization of the face, limited growth of the facial mass, and also, the reduction of the dentition. The hypothesis tested here is that skeletal and alveolar cephalometric factors would correlate with dental factors. In this French retrospective study, the following points were demonstrated:

- The type of missing tooth (3^rd^ molar or other) influences craniofacial morphological parameters;
- There is an association between agenesis and skeletal parameters in all three dimensions: vertical, transverse and sagittal;
- However, the parameters of this association differ according to the uni- or multivariate model used;

In light of the existing literature, these associations may have a genetic and phylogenetic rationale. The scope of research to be carried out in this field remains vast, with many hypotheses still to be tested.

## Supporting information

S1 Appendix Raw demographic and radiographic data from medical records. (XLSX)
